# The Role of Maintaining Nutritional Adequacy Status and Physical Activity in Onco-Nephrology: Not a Myth Anymore, but a Reality

**DOI:** 10.3390/nu17020335

**Published:** 2025-01-17

**Authors:** Francesco Trevisani, Matteo Paccagnella, Andrea Angioi, Francesco Fiorio, Matteo Floris, Andrea Pontara, Giuseppe Rosiello, Silvia Violante, Umberto Capitanio, Andrea Salonia, Francesco Montorsi, Arianna Bettiga

**Affiliations:** 1 Division of Experimental Oncology, Urological Research Institute (URI), IRCCS San Raffaele Scientific Institute, 20132 Milan, Italy; rosiello.giuseppe@hsr.it (G.R.); capitanio.umberto@hsr.it (U.C.); salonia.andrea@hsr.it (A.S.); montorsi.francesco@hsr.it (F.M.); bettiga.arianna@hsr.it (A.B.); 2 Department of Urology, IRCCS San Raffaele Scientific Institute, 20132 Milan, Italy; 3 A.O. Santa Croce e Carle, CTC, 12100 Cuneo, Italy; paccagnella.m@ospedale.cuneo.it (M.P.); violante.s@ospedale.cuneo.it (S.V.); 4 Department of Nephrology, Dialysis and Transplantation, G. Brotzu Hospital, 09134 Cagliari, Italy; andrea.angioi@aob.it (A.A.); matteo.floris@aob.it (M.F.); 5 Experimental Gastroenterology Lab, Division of Immunology, Transplantation and Infectious Disease, Department of Gastroenterology and Digestive Endoscopy, IRCCS San Raffaele Scientific Institute, 20132 Milan, Italy; fiorio.francesco@hsr.it; 6 Clinical Nutrition, IRCCS San Raffaele Scientific Institute, 20132 Milan, Italy; pontara.andrea@hsr.it

**Keywords:** physical activity, diet, onconephrology, personalized diet

## Abstract

**Background:** Physical Activity (PA) provides numerous biological and psychological benefits, especially for cancer patients. PA mitigates treatment side effects, influences hormones, inflammation, adiposity, and immune function, and reduces symptoms of anxiety, depression, and fatigue. This study evaluates the impact of PA on these positive outcomes. **Materials and Methods:** An observational retrospective study enrolled 81 patients: 31 with CKD stages II–V and 50 with CKD and urological malignancies. Baseline and 6-month follow-up visits included clinical (Iohexol, Creatinine, Cystatin C) and anthropometric parameters (Bioimpedance Analysis, body circumferences). Physical activity levels were assessed using the Rapid Assessment of Physical Activity (RAPA) test. Patients followed a Mediterranean-like diet with controlled protein intake (MCPD) and received PA improvement advice. Statistical analysis was performed using linear regression and Pearson’s Chi-Squared test with R programming. **Results:** Significant reductions in total adiposity and abdominal fat and improved body fluid distribution were observed. Post intervention, there was a 25.4% reduction in inactive individuals and an 88% increase in active lifestyles. Patients aged 75+ were more likely to be sedentary, indicating a need for increased professional attention. No correlation was found between increased PA and creatinine, cystatin, and eGFR values, but a positive correlation with GFR measured by iohexol clearance remained significant in multivariate analysis. Post intervention, regular PA engagement increased from 12.3% to 48% (*p* < 0.002). **Conclusions:** Incorporating PA and nutritional assessments into standard clinical care, supported by a collaborative nephrologist–nutritionist approach, can enhance the quality of life of CKD patients.

## 1. Introduction

Physical activity (PA) is increasingly being recognized for its significant benefits in managing chronic diseases, including chronic kidney disease (CKD) and cancer [1,2]. Aerobic and combined training (aerobic and resistance exercises) have been particularly noted for their ability to reduce symptoms of anxiety, depression, and fatigue, and to improve health-related quality of life (HRQoL) [1]. Patients undergoing cancer treatment often suffer from a range of debilitating symptoms and side effects, such as fatigue, muscle wasting, and psychological distress, which can adversely impact their quality of life [3]. Evidence suggests that regular physical activity can mitigate these effects by modulating various biological mechanisms, including hormone regulation, inflammation reduction, adiposity control, and immune function enhancement [2,4]. Moreover, recent studies suggest that physical activity may positively affect tumor biology, potentially reducing the risk of cancer recurrence and improving overall survival rates [5].

Recent meta-analyses have reinforced the role of PA in improving cancer survival rates and reducing recurrence. For example, Meyerhardt et al. (2017) found that colorectal cancer survivors who engaged in regular physical activity had a 24% lower risk of cancer-specific mortality [6]. Additionally, Schmitz et al. (2019) have emphasized that PA helps in reducing treatment-related side effects, including fatigue and bone density loss, highlighting the importance of integrating PA into cancer care protocols [7].

A recent scoping review by Arthuso et al. tried to elucidate the role of PA on renal cancer by identifying 17 articles published on the topic, deeming the evidence on the safety, feasibility, and benefits of PA for these patients to be limited and inconclusive and highlighting an urgent need for clinical exercise guidelines [8]. While the specific impact of PA on kidney cancer survival and prognosis is not well documented in the literature, there are some studies that have tried to analyze the role that a more active lifestyle may play for renal cancer incidence and prognosis. In 2016, Moore et al. gathered evidence suggesting that a higher level of PA is associated with a reduction in incidence risk greater than 20% for seven cancer types, among which we find kidney cancer [9]. In another and more recent systematic review and meta-analysis, Friedenreich et al. outlined how post-diagnosis PA was associated with lowered all-cause and cancer-specific mortality, with the biggest impact on the latter by more than 30% in all cancers taken into consideration, kidney cancer included [10]. Furthermore, in 2018, the World Cancer Research Fund International (WCRF) in its “Diet, nutrition, physical activity and kidney cancer report” outlined the importance of PA by stating in its main conclusions that higher body fat is a convincing cause for renal cancer and, therefore, means that maintaining a healthy body composition can have a positive impact on renal health [11].

In the context of CKD, the integration of a tailored physical activity regimen into patient care has been shown to have multifaceted benefits, including improved cardiovascular health, enhanced metabolic function, and reduced systemic inflammation [2]. A systematic review by Smart and Williams (2013) reported that regular aerobic exercise improved cardiorespiratory fitness and reduced blood pressure in CKD patients [12]. Given the heightened cardiovascular risk and the propensity for muscle wasting in CKD patients, incorporating structured exercise into treatment protocols could be particularly beneficial [1].

However, no clear evidence is currently present in the literature regarding the benefits of PA in onco-nephrological scenarios, where patients are affected by cancer and CKD at the same time [13]. This gap highlights the need for more research and targeted interventions. Emerging studies suggest potential benefits of tailored PA programs in improving clinical outcomes for onco-nephrology patients [14]; however, further proof must be sought to strengthen this correlation.

Therefore, our study aimed to explore the impact of a structured physical activity program within a multidisciplinary care model on clinical outcomes in a cohort of patients with CKD and a recent history of urological malignancies.

## 2. Materials and Methods

An observational retrospective study was conducted on a cohort of 81 consecutive patients who suffered CKD stage II–V with (50 patients) or without (31 patients) a history of urological malignancies, diagnosed within the previous 12 months, who were enrolled in a tertiary-care institution (IRCCS San Raffaele Hospital, Milan, Italy) between 2020 and 2022.

The exclusion criteria included age < 18 years, recurrence of acute kidney injury, end-stage renal disease requiring hemodialysis, concomitant chemotherapy, or immunotherapy, metastasis, and the absence of informed consent.

The study protocol required that all enrolled patients undergo a comprehensive nephrological and nutritional assessment at baseline and after 6 months (+/−2 months). Following the baseline evaluation, all participants underwent an initial nephrological and nutritional evaluation, and at their first visit received a Mediterranean-like controlled protein diet (MCPD) program, tailored to the specific stage of chronic kidney disease [15]. The patients received a diet with controlled protein (0.8–1.0 g/kg/die) and phosphorus (<1 gr/die) contents. A daily protein content of 1.0 gr/kg was assigned to all patients except those with metabolic alterations (hyperazotemia, metabolic acidosis, hyperkalemia, etc.) in accordance with ESPEN guidelines [16]. In this case, the protein content was reduced to 0.8 g/kg/die according to KDIGO guidelines [17].

The resting energy expenditure was determined by Mifflin–St Jeor for obese patients, while the Harris–Benedict equation was used for all the others. An activity factor of 1.4–1.6 was adopted to predict the total energy expenditure, since this value is indicated for adult patients with light physical activity [18]. At the first visit, patients were also given advice on how to increase their physical activity according to the recommendations from the American College of Sports Medicine (ACSM), the National Comprehensive Cancer Network (NCCN), and the Clinical Oncology Society of Australia (COSA), who advise engaging in 150 min of moderate-intensity aerobic exercise spread over 3–5 sessions per week, alongside resistance training at least twice a week [19,20,21]. The patients were educated about the meaning of moderate-intensity aerobic exercise (brisk walking and cycling) and resistance training (isometric floor body routines or with elastic bands) during the first outpatients’ visit.

The following clinical data were considered: age, gender, body mass index (BMI), type of cancer (kidney, bladder, prostate, urothelial), hypertension, diabetes and medical therapy (ACE inhibitors (ACEi), angiotensin II receptor blockers (ARBs), calcium antagonists, beta-blockers, and diuretics.

Clinical blood tests were performed to evaluate renal function, with serum creatinine, cystatin C and urea measured at baseline and at follow-up, following routine blood exams. GFR was estimated at each time point using the creatinine-based estimated glomerular filtration rate (eGFR) formula CKD-EPI (2021) [22], while gold-standard measurements using Iohexol plasma clearance were employed in selected patients to eliminate possible bias linked with sarcopenia for eGFR that might lead to an underestimation of renal function [23].

CKD stages were defined using the estimated Glomerular Filtration Rate (eGFR) with CKD-EPI formula, 2021, according to K-DIGO guidelines, 2023. In line with these guidelines, CKD stage IIIa was defined with an eGFR lower than 60 mL/min but higher than 45 mL/min; CKD stage III b with an eGFR lower than 45 mL/min but higher than 30 mL/min; and CKD IV stage with an eGFR lower than 30 mL/min but higher than 15 mL/min.

The study received the approval of the Institutional Ethical Committee (San Raffaele Hospital, Milan, Italy, Protocol Code/Acronym URBBAN, approval date 3 March 2014), and informed consent was obtained from all of the patients included in the study. All the experimental procedures involving human biological material were carried out in compliance with the approved guidelines and according to good clinical practice.

### 2.1. Assessment of Nutritional Status and Physical Activity

In order to assess the patients’ nutritional status, anthropometric measurements were performed: for each patient, we collected body weight and height in order to calculate the corresponding Body Mass Index (BMI), Waist Circumference, and Waist–Hip ratio. A Professional Body Impedance Analyzer (BIA-Dex, Mascaretti srl, Piacenza, Italy) was used to study the patients’ body composition: we collected data for Phase Angle (PA), Body Cellular Mass (BCM)—Height2 ratio, Extracellular mass (ECM)—BCM ratio, Extracellular water (ECW)—Intracellular Water (ICW) ratio, TotalWater (TBW%), Fat Mass (FM)—Height2 ratio and Free Fat Mass (FFM)—Height2 ratio, and Mid-Upper Arm Muscle Circumference (MAMC). MAMC was calculated using the formula MAMC = Mid-arm Circumference − (3.1415 × Triceps Skinfold Thickness (TSF)) [24]. The Malnutrition Universal Screening Tool (MUST) was used to identify malnutrition. The Rapid Assessment of Physical Activity (RAPA) test was employed to evaluate the physical activity levels in our cohort of patients. The RAPA test is a validated, user-friendly tool designed to evaluate physical activity levels in older adults. It comprises a series of questions that address both the frequency and intensity of physical activity (RAPA 1), as well as flexibility and strength-training activities (RAPA 2). In our cohort, the RAPA test was instrumental in capturing the spectrum of physical activity behaviors, offering critical insights into the patients’ overall physical activity levels and identifying those at risk of sedentary lifestyles [25].

### 2.2. Statistical Analysis

Comparisons among values of each variable before and after dietary intervention were performed with the Wilcoxon signed-rank test for repeated measures. Differences in categorical variables were analyzed with the χ^2^-test or Fisher’s exact test. Correlations were realized with Spearman’s rank correlation coefficient. Linear regression analysis was used to compare variables with RAPA-1 as the target variable. Multinomial logistic regression was performed to test variables using RAPA-2 as the target variable. All analyses were assessed using JMP^®^, Version 17 Pro. SAS Institute Inc., Cary, NC, USA, 1989–2024. In all tests, a *p* < 0.05 was regarded as significant.

## 3. Results

### 3.1. Clinical and Demographic Findings of the Patients

The study enrolled a total of 81 patients, of whom 50 (61.7%) had a history of urological malignancies (oncology group) and 31 (38.3%) served as controls affected with CKD but without malignancies (Table 1a). The cohort’s mean age was 70.9 years (IQR: 63–76), with no significant differences between groups (71.9 vs. 67.9 years, *p* = 0.441). Male participants were prevalent (79.5%), and there was a higher proportion of males in the oncology group than the control group (82.0% vs. 67.7%, *p* = 0.142), though this difference did not reach statistical significance.

Body composition analysis revealed a comparable median BMI (oncology: 26.0 kg/m^2^ vs. control: 25.3 kg/m^2^, *p* = 0.972); approximately 44.4% of the overall cohort was classified as having a healthy weight, 32.1% as overweight, and 23.5% as obese. Remarkably, the prevalence of advanced CKD stages was higher in the control group; CKD stage G5 was observed in 16.1% of control patients compared to 2% in the oncology group (*p* = 0.062), whereas stages G3a and G3b predominated in the oncology cohort, but without statistical significance.

Hypertension was the most common comorbidity, affecting 76.5% of the entire cohort, with similar prevalence across both groups (oncology: 74.0%, control: 80.6%, *p* = 0.682). Diabetes was present in 11.1% of the cohort and was more frequent in the oncology group (14.0%) compared to controls (6.4%), though this difference was not statistically significant (*p* = 0.492).

The oncology cohort comprised 55 distinct urological cancer cases. Kidney cancer accounted for nearly half of the malignancies (26/55; 47.2%), followed by bladder cancer (17/55; 30.9%) and prostate cancer (6/55; 10.9%). Surgical interventions varied by tumor type, with radical nephrectomy performed in 16 kidney cancer cases (61.5%) and cystectomy in 14 bladder cancers (82.4%). Partial nephrectomy (38.5%) and TURBK (bladder and urothelial cancers; 9.4%) were less common (Table 1b).

### 3.2. Improvement in Physical Activity Levels in Nephrological Patients with or Without Oncological History

**Figure 1:**
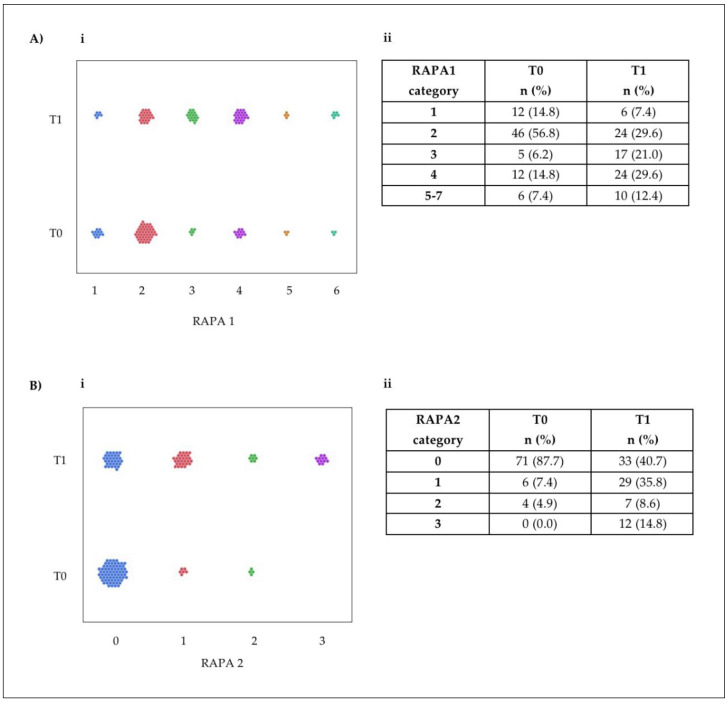
Data representation for patient-reported RAPA test scores in the cohort: visual representation of the data: (**A**) (**i**) RAPA 1 scores; (**B**) (**i**) RAPA 2 scores. Each dot represents one patient. (**A**) (**ii**) RAPA 1 scores; (**B**) (**ii**) RAPA 2 scores. *n* indicates the number of patients who reported a given score on the scale.

The six-month intervention markedly improved physical activity performance across the cohort, as illustrated in Figure 1. At baseline (T0), most patients exhibited low physical activity levels. Specifically, 77.7% of participants were classified as sedentary or underactive (RAPA1 score ≤ 3), with only 7.4% achieving active status (RAPA1 score ≥ 6). Following the intervention (T1), there was a significant shift toward higher activity levels, with an 88% increase in participants classified as regularly active and a 25.4% reduction in highly inactive. This progression was reflected in the mean RAPA1 score, which increased significantly from 2.47 at baseline to 3.17 at follow-up (*p* < 0.0001).

The improvement in strength and flexibility activities, captured by RAPA2 scores, was equally pronounced. At T0, only 12.3% of patients engaged in these activities regularly, while 48% reported regular participation after the intervention—an increase of nearly fivefold (*p* < 0.002). Category shifts in RAPA2 scores showed a notable reduction in completely inactive individuals (Category 0) and significant increases in Categories 1–3, representing progressively higher levels of engagement in strength and flexibility exercises.

### 3.3. Improvement in Resistance Physical Activity in Patients Prone to Increasing Their Aerobic Activity Levels

The impact of resistance exercise on CKD patients has gathered increasing attention and may be an ideal choice for enhancing disease prognosis. This type of physical training does not necessarily involve very intense activities that could harm the body; it includes various forms, and the use of instruments such as resistance bands and dumbbells may improve its efficacy. Additionally, resistance exercise can improve metabolic parameters with less energy consumption compared to other types of training, making it more feasible for CKD patients, especially for those with poor cardiopulmonary function. Moreover, it can effectively alleviate the complications of CKD, including metabolic syndrome and sarcopenic obesity, and reduce related biomarkers [26].

Table 2 shows that patients who did not incorporate muscle strengthening and/or flexibility activities into their lifestyle (RAPA 2 score 0) were also less likely to increase their aerobic activity levels. The strongest correlation between the increase in RAPA 2 levels and RAPA 1 levels after the nutritional–nephrological intervention, obtained by setting a linear mixed model with RAPA 1 as the target and RAPA 2 as the fixed effect, was achieved for a RAPA 2 score of 3. Patients who significantly increased their aerobic activity levels included muscle strengthening and flexibility in their weekly physical activity. However, muscle strengthening activity was the most critical parameter correlating with the patient’s aerobic activity level (RAPA 2 score 1). Patients who performed only muscle strengthening increased their aerobic physical activity levels compared to those who also performed flexibility activities. Thus, flexibility activities did not appear to influence the increase in patients’ aerobic physical activity levels.

### 3.4. Onco-Urological History Is Not a Contrasting Factor for Patients to Adopt an Inactive Lifestyle

**Figure 2:**
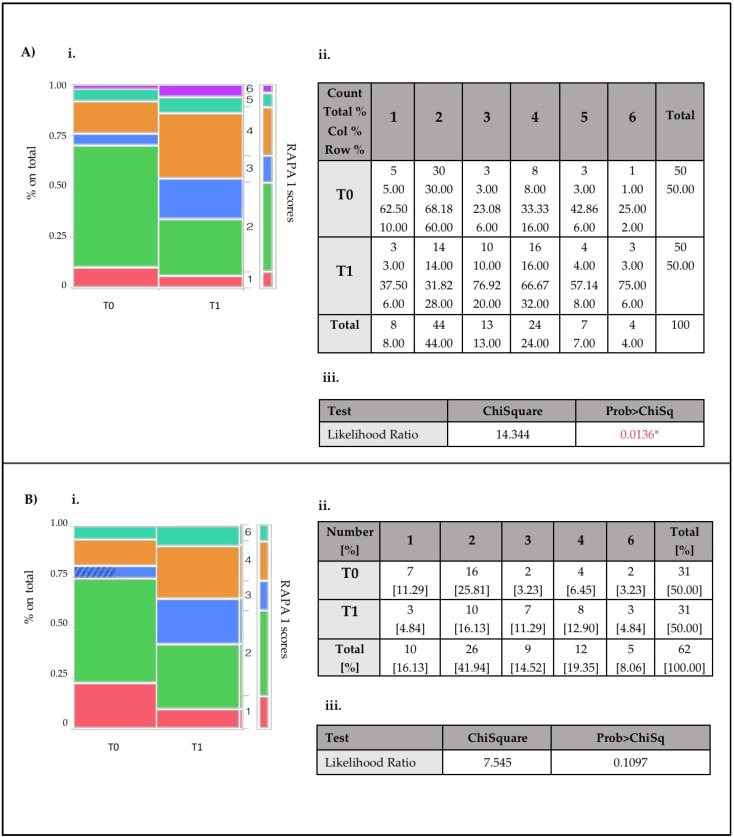
(**i**) Mosaic plot illustrating the changes in RAPA 1 scores for the onco-nephropathic patient (**A**) and nephropathic patient (**B**) subsets between T0 and T1, expressed as percentages of the total cohort. (**ii**) Contingency table providing the numerical breakdown corresponding to the mosaic plot. (**iii**) Likelihood ratio test results with associated Chi-Square statistics and *p*-value (**iii**). Warning: 20% of cells have an expected count less than 5, so the Chi-Square is suspect. * Indicates statistical significance for *p* = 0.05.

**Figure 3:**
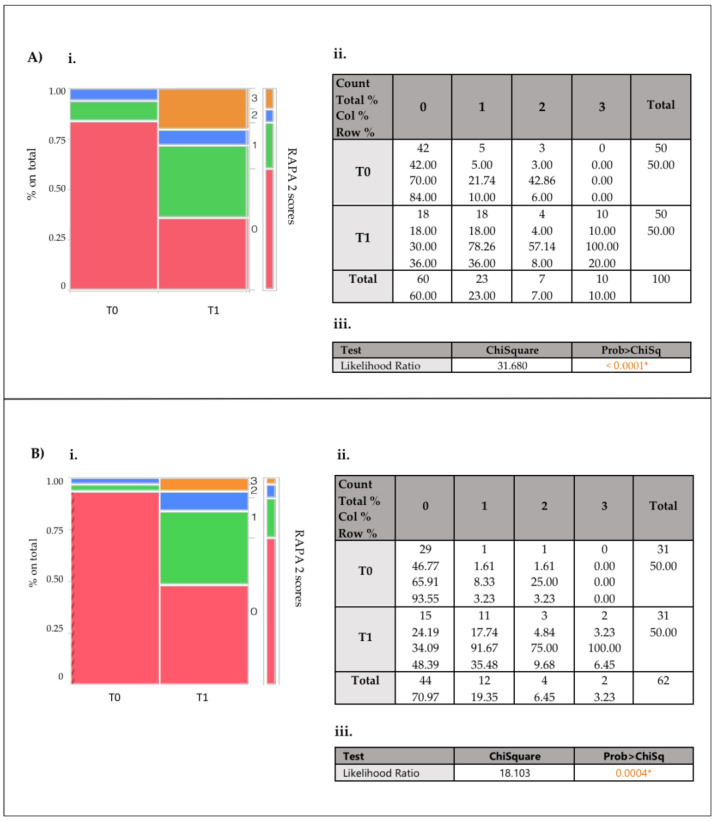
(**i**) Mosaic plot illustrating the changes in RAPA 1 scores for the onco-nephrological patient (**A**) and nephropathic patient (**B**) subsets between T0 and T1, expressed as percentages of the total cohort. (**ii**) The contingency table provides the numerical breakdown corresponding to the mosaic plot. (**iii**) Likelihood ratio test results with associated Chi-Square statistics and *p*-value (**iii**). Warning: 20% of cells have an expected count of less than 5, so the Chi-Square is suspect. * Indicates statistical significance for *p* = 0.05.

The intervention revealed significant improvements in physical activity levels (Figure 2 and Figure 3); in particular, for the onco-nephrological patients, results indicated that at T0, more than two-thirds of patients exhibited a sedentary lifestyle, characterized by a RAPA 1 score of less than or equal to 3 (76% of the population) and by a RAPA 2 score of 0 (84% of the population), indicating that patients performed low aerobic activity and did not incorporate any muscle strengthening and/or flexibility activities into their lifestyle. The results are even more alarming for patients with kidney disease alone: only six and two out of thirty patients perform regular aerobic and resistance activities, respectively.

In the patients with cancer and concomitant kidney disease, a marked redistribution across RAPA1 categories was observed, with significant reductions in sedentary participants (Score 1) and increases in those classified as underactive or active (from score 1 to score 2: *p* = 0.0136; from score 1 to score 4: *p* = 0.0163) (Figure 2A). The patients with kidney disease alone showed similar trends, with a reduction in sedentary individuals and an increase in active participants, but without achieving statistical significance (*p* = 0.1097) (Figure 2B).

Improvements in RAPA2 scores were significant in both groups, with dramatic reductions in completely inactive individuals (Score 0) and increases in different resistant activity levels (Scores 1 and 3). These changes were particularly relevant in the onco-nephrological patients, with significant transitions from inactivity to higher activity categories (from score 0 to score 1: *p* = 0.0003; from score 0 to score 3: *p* < 0.0001) (Figure 3A). Nephrological patients also demonstrated substantial improvements, transitioning from inactivity to higher activity categories (from score 0 to score 1: *p* = 0.0002; from score 0 to score 3: *p* = 0.0001) (Figure 3B).

### 3.5. Age Is a Contrasting Factor for Patients to Adopt an Inactive Lifestyle

**Figure 4:**
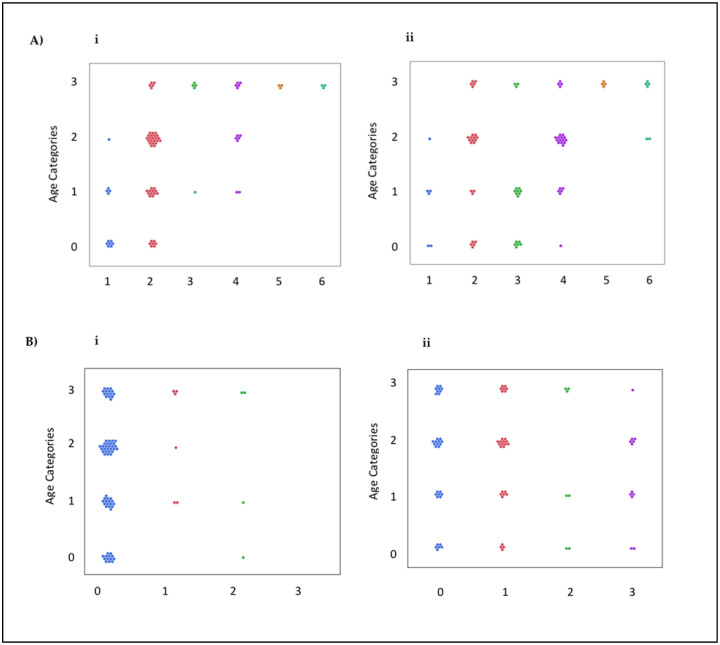
Visual representation of the response to physical activity advice for different age categories: 0: <1943; 1: 1943–1947; 2: 1948–1957; 4: 1957. Figure (**A**) (**i**) shows the RAPA 1 scores at T0; Figure (**A**) (**ii**) shows the RAPA 1 scores at T1. Figure (**B**) (**i**) shows the RAPA 2 scores at T0; Figure (**B**) (**ii**) shows the RAPA 2 scores at T1. Each dot represents one patient.

Emerging data, regardless of age, demonstrate that CKD patients have a high prevalence of low physical activity and frailty that is similar to or higher than that in the general population of elderly adults [27]. When stratified by age groups (Figure 4), we found that 39.5% of people aged 74 years and younger had a sedentary lifestyle (RAPA score ≤ 3), with a higher prevalence among people older than 65 years (71.8%). In addition, our study identified people aged 75 years or older as a subpopulation that was more likely to have a sedentary lifestyle among patients with CKD (31 out of 33 subjects were identified as sedentary or underactive), indicating that increased attention from healthcare professionals may be needed. Only 18 subjects, corresponding to 22% of the entire cohort, had an active lifestyle: 61% of people aged 65 years and younger and only 11.1% aged 75 years and older.

Considering strength or balance exercises, we found that 87.6% of people in the entire cohort, equally distributed by age group, did not do any regular strength or balance training combined with endurance.

Our data are in line with those present in the literature. Older adults may have difficulty meeting the traditional 150 min (min)/week of moderate to vigorous physical activity guidelines (US Department of Health and Human Services, 2018. Physical Activity Guidelines for Americans, 2nd edition. US Department of Health and Human Services, Washington, DC). The statistics reported that for older adults, only 38% of those aged 50–64 years and 15% of those 75 or older meet both recommended aerobic and muscle-strengthening physical activity guidelines [28].

Following the counseling intervention, there was a 56% reduction in sedentary and underactive individuals and a 47% increase in the number of patients with moderately active or active lifestyles, indicated by a RAPA 1 score of 4 or higher: 35% of people aged 65 years and younger and only 17.6% of those older than 75 years. Regarding strength or balance exercises, we found that 38% of people in the entire cohort continued to abstain from regular training, but 33.3% of patients, equally distributed by age group, carried out activities to increase muscle strength, such as lifting weights or calisthenics, once a week or more. Strength or balance training appears to be unaffected by patients’ age; on the other hand, we found that younger subjects predominately carried out resistance training.

### 3.6. Association of a Diet-and-Physical-Activity Combined Approach and the Improvement of Anthropometric Parameters

Table 3 collects the anthropometric indices of our patients measured by BIA before and after the diet-and-physical-activity combined approach. Our data showed a significant reduction in total adiposity and abdominal fat; there were substantial changes in BMI in the surrogate measurement of abdominal obesity (waist circumference) and FMh2. BIA assessments such as PA and FFM correlate with nutritional status and survival in patients with nephropathic and oncologic disease. Analyzing the variation for the entire population, we observed an overall improvement not only in PA and FFMh2, which indicates advancement in the nutritional status of the patients, but in most of the anthropometric parameters considered. A new parameter for assessing nutrition status, in addition to the commonly used PA, is the extracellular mass-to-body cell mass ratio (ECM/BCM). The BCM is the overall cell mass, which is metabolically active and therefore responsible for the metabolic rate; the ECM includes interstitial water (ascites, pleural effusion, and so on) and connective tissues such as collagen, elastin, skin, tendons, and bone. In healthy individuals, the BCM is always distinctly higher than the ECM and, as a consequence, their ratio is less than 1 [29]. Increased BCM index and a reduced ECM/BCM ratio indicate a healthy fluid balance and absence of malnutrition in most of the population. In our study, by combining diet and physical activity in nephropathic patients with or without urological cancer history, ECM/BCM was lowered from 0.91 to 0.87, signaling improved nutrition status. The variation in the MAMC parameter did not show any significance in this analysis. In terms of fluid balance, we observed an increase in TBW% and a decrease in ECW/ICW ratio, indicating an improvement in body fluid distribution between the intracellular and extracellular compartments, consisting of muscle cell mass (i.e., myofibers) and plasma and interstitial fluids, respectively.

Univariate analysis demonstrated significant associations between RAPA1 scores and key anthropometric and physiological parameters (Table 4 and Table S1). Among these, PA (estimate: 0.851, *p* < 0.001), BCM/h^2^ (estimate: 0.321, *p* < 0.001), FFM/h^2^ (estimate: 0.184, *p* < 0.001), and TBW% (estimate: 0.072, *p* < 0.001) showed strong positive correlations. Conversely, RAPA1 scores were negatively correlated with ECM/BCM (estimate: −4.846, *p* < 0.001), ECW/ICW (estimate: −3.943, *p* < 0.001), and FM/h^2^ (estimate: −0.140, *p* < 0.001). These findings suggest that increased physical activity reduces extracellular fluid excess and fat mass while enhancing intracellular water content and lean tissue metrics.

On the other hand, BMI (*p* = 0.482) and waist circumference (*p* = 0.466) were not significantly associated with RAPA1 scores, indicating that changes in overall activity levels may not directly impact these general measures of adiposity.

### 3.7. Association of a Diet-and-Physical-Activity Combined Approach and Changes in Nephrological Scenario

The six-month intervention with the combined approach demonstrated stability in kidney function and significant improvements in metabolic parameters across the cohort. Kidney function, assessed first with serum creatinine and then eGFR (CKD-EPI 2021 equation), showed no statistically significant change from baseline to follow-up (median serum creatinine: 1.615 mg/dL [IQR: 1.41–2.14] vs. 1.58 mg/dL [IQR: 1.38–2.31], *p* = 0.46375; median eGFR: 44.0 mL/min/1.73 m^2^ [IQR: 33.0–52.25] vs. 45.0 mL/min/1.73 m^2^ [IQR: 37.0–47.0], *p* = 0.74356). Iohexol clearance corroborated these findings, with no significant variation during the temporal interval considered (Table 5).

In contrast to the stability of GFR metrics, the intervention yielded a notable reduction in serum urea levels (median: 61.5 mg/dL [IQR: 42.75–75.5] vs. 48.5 mg/dL [IQR: 40.5–66.0], *p* = 0.00516). This decrease may reflect an improved protein metabolism and compliance with the Mediterranean-like controlled protein diet (MCPD), suggesting a metabolic benefit without imposing additional “alimentary” stress.

The univariate analysis examining the relationship between physical activity (RAPA1 scores) and renal function metrics revealed that while no significant associations were found between RAPA1 scores and eGFR (*p* = 0.661), serum creatinine (*p* = 0.751), or cystatin C levels (*p* = 0.263), a significant positive correlation emerged between physical activity levels and mGFR using iohexol clearance (estimate: 0.06, *p* = 0.004) (Table 6).

## 4. Discussion

### 4.1. The Role of Physical Activity in Onco-Nephrology

Physical activity (PA) is increasingly recognized as a cornerstone of care for patients with chronic kidney disease (CKD) and cancer, offering multifaceted benefits that address physical, physiological, and psychological challenges. These benefits are particularly critical given the dual burden of CKD and cancer, which exacerbates sarcopenia, inflammation, and cardiovascular risks, all of which contribute to a decline in quality of life (QoL) and functional capacity. Targeted interventions are essential to improve these outcomes, enhance survival rates, and reduce the burden on healthcare systems.

With the rise of onco-nephrology, a field dedicated to addressing the complex interplay between oncological and renal conditions, there is a pressing need for new combined strategies. Multidisciplinary approaches must integrate personalized medical care with physical activity programs and dietary interventions to mitigate the incidence of acute kidney injury (AKI) and the progression of CKD during oncological treatments [30]. AKI, often induced by nephrotoxic chemotherapeutic agents, and CKD are significant contributors to morbidity in cancer patients, further highlighting the urgency of effective prevention strategies [31,32].

One of the primary challenges in treating onco-nephrological patients is their increased susceptibility to sarcopenia—a condition characterized by significant muscle mass and function loss, often aggravated by the metabolic stress of cancer and CKD [33]. Sarcopenic patients are less capable of tolerating medical treatments, including chemotherapy and surgical interventions, which undermines the efficacy of these therapies. Studies have demonstrated that sarcopenia not only reduces tolerance to treatment but is also independently associated with worse outcomes, including higher mortality rates [34,35].

Furthermore, inactivity is prevalent among this patient population. Many CKD and cancer patients lead sedentary lifestyles, which compounds their vulnerability and exacerbates the cycle of physical decline. Regular physical activity, particularly tailored to the patient’s condition, has been shown to mitigate these effects by preserving muscle mass, improving cardiovascular fitness, and enhancing overall functional capacity [36]. However, despite these benefits, only a small percentage of patients, particularly older adults, meet the recommended guidelines for physical activity. For instance, only 38% of adults aged 50–64 and 15% of those aged 75 or older meet aerobic and muscle-strengthening guidelines, emphasizing the need for targeted interventions [37].

Our innovative study aimed to investigate the transformative potential of combining dietary management and structured physical activity as a multidisciplinary approach to enhance clinical outcomes in CKD patients with or without urological malignancies. To do that, we enrolled a consecutive cohort of 81 patients affected by CKD in a tertiary care institution, of whom 50 (61.7%) had a concurrent history of urological malignancies. We proposed a six-month intervention to implement the level of physical activity in all of them combined with a tailored dietary regime based on renal function. Nutritionists’ and nephrologists’ evaluations were performed at baseline and after 6 months, with all clinical and laboratory data reported.

One of the study’s most significant findings was the marked reduction in sedentary behavior post intervention, as evidenced by RAPA 1 scores. Before the intervention, 77.7% of participants were classified as sedentary or underactive. After six months, this percentage significantly dropped, with an 88% increase in those achieving active status. These results align with the existing literature, where tailored physical activity interventions have demonstrated similar success in reducing inactivity among sedentary CKD populations [36].

This first highlight is remarkable because once patients are educated about the importance of engaging in physical activity, they are strongly willing to adopt it, showing no hesitation and fully understanding its therapeutic value. This concept is particularly noteworthy because it suggests that the prevalent inactivity observed in such populations may not stem from a lack of capability but rather from insufficient communication regarding the role of physical activity as a core component of their treatment plan. By addressing this gap in patient education, healthcare providers can play a pivotal role in empowering patients to incorporate physical activity into their daily lives, thereby improving both their clinical outcomes and quality of life.

A second important aspect that we have investigated is the impact of incorporating resistance and flexibility activities on enhancing aerobic physical activity levels in CKD patients. As demonstrated in the results, patients who did not engage in muscle-strengthening or flexibility activities (RAPA 2 score 0) were also less likely to increase their aerobic activity levels (RAPA 1). The linear mixed model analysis, with RAPA 1 as the dependent variable and RAPA 2 as the fixed effect, revealed that the most substantial improvement in RAPA 1 scores was associated with patients who achieved a RAPA 2 score of 3. These individuals incorporated muscle-strengthening and flexibility exercises into their weekly routines, demonstrating the synergistic effects of combining these activities.

However, the analysis also identified muscle-strengthening activities as the most important factor correlating with aerobic physical activity. Patients who engaged solely in muscle-strengthening exercises (RAPA 2 score 1) showed comparable improvements in RAPA 1 scores to those who performed both muscle strengthening and flexibility activities. This suggests that while flexibility exercises complement resistance training, they do not influence aerobic activity levels independently. Therefore, prioritizing muscle-strengthening activities in exercise programs may yield the most significant benefits for CKD patients, increasing their overall physical activity and improving metabolic and functional outcomes.

A significant redistribution across RAPA1 categories was observed in patients with cancer and concomitant kidney disease, reflecting notable reductions in sedentary behavior and transitions to more active lifestyles. Specifically, there was a marked decrease in participants classified as sedentary (Score 1) and corresponding increases in those classified as underactive or active (Score 2 and Score 4). These changes were statistically significant, with transitions from Score 1 to Score 2 (*p* = 0.0136) and from Score 1 to Score 4 (*p* = 0.0163) (Figure 2A). This suggests that the intervention was particularly effective in encouraging onco-nephrology patients to engage in higher physical activity.

Improvements in RAPA2 scores, which reflect resistance and flexibility activity levels, were significant in both groups. A dramatic reduction in completely inactive individuals (Score 0) was observed, accompanied by increases in higher resistance activity levels (Scores 1 and 3). These changes were particularly pronounced in onco-nephrology patients, with significant transitions from inactivity to higher activity levels (Score 0 to Score 1: *p* = 0.0003; Score 0 to Score 3: *p* < 0.0001) (Figure 3A). Similarly, nephrological patients demonstrated substantial improvements in resistance activity levels, with significant transitions from Score 0 to Score 1 (*p* = 0.0002) and from Score 0 to Score 3 (*p* = 0.0001) (Figure 3B).

These findings highlight the efficacy of the intervention in promoting physical activity, particularly resistance exercises, across both patient populations. The more robust response observed in onco-nephrology patients may reflect a heightened awareness of the importance of physical activity in mitigating the dual burdens of cancer and kidney disease. The transition from inactivity to higher activity levels, particularly in resistance exercises, is a critical outcome, given the well-documented benefits of resistance training in improving muscle mass, physical function, and overall quality of life in these populations.

A Cochrane systematic review suggested that exercise, when performed for more than 30 min three times per week, can significantly improve nutritional status, blood pressure, physical well-being, and quality of life in CKD patients [37]. However, despite the well-documented benefits of exercise, CKD patients remain predominantly physically inactive. Fatigue and concerns about safety are frequently cited as primary barriers to initiating or maintaining regular physical activity. Interestingly, internal motivation to improve health and support from healthcare professionals has been identified as a strong driver for exercise adoption among this population [38]. This finding underscores the importance of educating patients about the benefits of exercise and equipping healthcare providers with the tools and training necessary to assess, counsel, and encourage physical activity as a critical component of CKD management [39].

A recent national survey of nephrologists in Saudi Arabia revealed that while most physicians acknowledge the role of exercise in CKD management and express a positive attitude towards recommending it, validated exercise programs designed explicitly for CKD patients still need to be made available. Similarly, a cross-sectional study of 62 nephrology centers in Italy published in 2024 highlighted the significant gap between awareness of the benefits of physical activity and its implementation in clinical practice. Although 93% of nephrologists agreed on the scientific evidence supporting the benefits of exercise for CKD patients, only 26% of centers offered exercise programs, primarily targeting dialysis patients. Furthermore, 63% of centers rarely or never assessed or recommended physical activity during patient care. Among the various forms of exercise, aerobic activities were considered the most beneficial by 81% of nephrology centers, followed by muscle strengthening (12%), muscle stretching (8%), and endurance exercises (3%).

The situation is similarly challenging in the oncological setting. Barriers such as lack of motivation, fear of injury, and limited access to tailored exercise programs hinder physical activity adoption among cancer patients. Despite the consolidated evidence supporting the long-term health benefits of physical activity, only 7% of cancer patients in Italy are sufficiently active [40]. Perception is crucial in determining physical activity behaviors in nephrology and oncology contexts. A positive attitude, defined as the perception of a behavior as beneficial, has been linked to greater adherence and maintenance of physical activity. Studies have consistently reported that oncological patients face barriers related to treatment side effects and generally perceive physical activity positively. This perception is associated with multiple benefits, including improved general health, better management of treatment-related side effects and chronic conditions, reduced stress, and alleviation of depression [40,41].

Healthcare professional advice is another critical factor influencing physical activity behaviors. A large-scale study involving 15,254 cancer survivors demonstrated that patients who recalled receiving physical activity advice during their treatment were significantly more likely to maintain higher levels of physical activity 2–3 years post-treatment than those who did not [42]. However, qualitative research has identified gaps in both professional knowledge and patient awareness regarding the importance of physical activity for reducing disease recurrence and promoting long-term health. This knowledge gap may partly explain the low physical activity levels observed in cancer patients with or without kidney disease in our cohort [43].

### 4.2. Dietary Regimen and Physical Activity Combination

The authors also investigated the relationship between body composition and physical activity in depth. Our data revealed a significant reduction in total adiposity and abdominal fat, as evidenced by marked improvements in BMI, waist circumference, and FM/h^2^. Bioelectrical impedance analysis (BIA) assessments, such as phase angle (PA) and fat-free mass adjusted for height (FFM/h^2^), are well-established indicators of nutritional status and survival in patients with nephrological and oncological conditions. Across the entire study population, we observed significant improvements in PA and FFM/h^2^—indicative of enhanced nutritional status—and positive changes in most of the anthropometric parameters analyzed.

In addition to PA, our study incorporated the extracellular mass to body cell mass ratio (ECM/BCM) as a parameter for assessing nutritional status. BCM represents the metabolically active cell mass responsible for the body’s metabolic rate. At the same time, ECM includes interstitial water (e.g., ascites, pleural effusions) and connective tissues such as collagen, elastin, skin, tendons, and bone. In healthy individuals, BCM is distinctly higher than ECM, resulting in an ECM/BCM ratio of less than 1. This balance is a hallmark of good nutritional health [44]. An increased BCM index and a reduced ECM/BCM ratio indicate a healthy fluid balance, a decrease in sub-chronic inflammation, and the risk of malnutrition, characteristics observed in most population studies.

In our intervention, which combined dietary modifications and physical activity for nephropathic patients, both with and without a history of urological cancer, the ECM/BCM ratio decreased from 0.91 to 0.87. This reduction reflects a significant improvement in nutritional status and cellular health.

Regarding fluid balance, our findings indicated a notable increase in total body water percentage (TBW%) alongside a decrease in the extracellular to intracellular water ratio (ECW/ICW). These shifts suggest an improvement in the distribution of body fluids between the intracellular and extracellular compartments. This redistribution likely reflects enhanced muscle cell mass (e.g., myofibers) and reduced excess extracellular fluids, such as plasma and interstitial fluids. However, the mid-arm muscle circumference (MAMC) parameter did not show significant variation in this cohort, suggesting that further investigation into muscle-specific adaptations may be warranted. These findings underscore the efficacy of combined dietary and physical activity interventions in improving nutritional status and fluid balance in this patient population.

### 4.3. Renal Function and Physical Activity Interventions

The six-month intervention using the combined approach demonstrated notable metabolic improvements while maintaining stability in kidney function across the cohort. Renal function, assessed through serum creatinine and estimated glomerular filtration rate (eGFR) using the CKD-EPI 2021 equation, remained consistent from baseline to follow-up. Median serum creatinine values showed no statistically significant change (1.615 mg/dL [IQR: 1.41–2.14] vs. 1.58 mg/dL [IQR: 1.38–2.31], *p* = 0.46375), nor did median eGFR values (44.0 mL/min/1.73 m^2^ [IQR: 33.0–52.25] vs. 45.0 mL/min/1.73 m^2^ [IQR: 37.0–47.0], *p* = 0.74356). These findings were further corroborated by the stability observed in iohexol clearance measurements, indicating no significant variation over the intervention period (Table 5). These results underscore the safety of the intervention, demonstrating that it did not impose additional stress on renal function, a critical consideration for this patient population.

While renal metrics remained stable, the intervention yielded a significant reduction in serum urea levels, which decreased from a median of 61.5 mg/dL [IQR: 42.75–75.5] to 48.5 mg/dL [IQR: 40.5–66.0] (*p* = 0.00516) [45]. This finding is particularly relevant as it suggests enhanced protein metabolism, likely attributable to adherence to the Mediterranean-like controlled protein diet (MCPD). The MCPD is designed to optimize protein intake within a moderate range, reducing metabolic burden while ensuring adequate nutritional support. The reduction in urea levels highlights the metabolic benefits of the intervention without imposing additional “alimentary” stress on renal function.

These results emphasize the intervention’s dual efficacy in maintaining renal stability while achieving metabolic improvements, particularly in urea metabolism. This balance is essential for CKD patients, who are often at risk of both renal deterioration and metabolic derangements. Future studies should explore the long-term sustainability of these effects and their implications for clinical outcomes, including quality of life and disease progression.

The univariate analysis exploring the relationship between physical activity levels, as measured by RAPA1 scores, and renal function metrics revealed nuanced findings. While no significant associations were observed between RAPA1 scores and conventional renal markers such as eGFR (*p* = 0.661), serum creatinine (*p* = 0.751), or cystatin C levels (*p* = 0.263), a notable positive correlation emerged between RAPA1 scores and measured glomerular filtration rate (mGFR) determined via iohexol clearance (estimate: 0.06, *p* = 0.004) (Table 5) [46]. This finding underscores the potential of physical activity to influence renal health through mechanisms not fully captured by traditional biomarkers.

The lack of significant associations with eGFR and serum creatinine may reflect the inherent limitations of these markers in detecting subtle changes in renal function. Both eGFR and serum creatinine are influenced by non-renal factors such as age, muscle mass, and nutritional status, which could obscure their sensitivity to improvements linked to physical activity [47,48]. Cystatin C is stably expressed in all nucleated cells and is used clinically to assess renal function because of its relatively small molecular weight and ease of detection. The fact that it is less affected by height, gender, age, and the patient’s muscle mass makes it an ideal marker of the glomerular filtration rate. However, our study, similar to creatinine, failed to show a significant relationship with physical activity in this cohort. In recent years, the involvement of cystatin C in the antitumor immune responses and an elevated expression of Cys C in cancer tissues have been reported, above all in urogenital malignancy, and seem to be correlated with prolonged time of chemotherapy [49,50]. This highlights the need for more precise and dynamic measures of renal function, such as mGFR via iohexol clearance, which demonstrated a statistically significant association with increased physical activity levels.

The positive correlation between mGFR and RAPA1 scores suggests that increased physical activity may contribute to maintaining or improving renal filtration capacity. Physical activity has been shown to enhance systemic hemodynamics, reduce inflammation, and improve vascular function, which are critical for preserving kidney health [31,36]. In particular, aerobic exercise has been associated with improved renal perfusion and oxygenation, potentially explaining this study’s observed relationship with mGFR [51]. This finding aligns with existing evidence supporting the protective effects of physical activity on renal function, particularly in patients with early-stage CKD or at high risk of disease progression [52].

Moreover, the significant association between physical activity and mGFR highlights the role of tailored interventions in optimizing outcomes for CKD patients. While traditional renal markers may not adequately capture the benefits of physical activity, mGFR provides a more direct and accurate assessment of glomerular filtration, underscoring the value of incorporating advanced diagnostic tools in clinical and research settings. These results also emphasize the need for personalized exercise programs that address CKD patients’ unique physiological and functional limitations, maximizing their potential to improve renal and overall health outcomes [30].

### 4.4. Limitations

This study has several limitations that should be considered when interpreting its findings. First, it was conducted on a relatively small retrospective cohort of patients, which may limit the generalizability of the results. The retrospective design also introduces potential biases in data collection and analysis, which could affect the reliability of the conclusions.

Secondly, the absence of a control group represents a significant limitation. With a group of patients who followed the dietary intervention alone, isolating the specific effects of physical activity on the observed outcomes is easier. A control group is necessary to establish a direct causal relationship between exercise and the clinical improvements observed in the cohort.

Thirdly, patients were not consistently monitored by physiotherapists during their exercise routines. This lack of professional supervision may have impacted the uniformity and effectiveness of the physical activity intervention. Without direct oversight, it is challenging to verify adherence to prescribed exercises or the accuracy of their execution, which could influence the intervention’s overall success.

Lastly, the study did not use objective tools such as dynamometers or similar devices to measure muscle strength. The absence of these instruments limits the ability to quantify changes in physical strength and assess improvements in sarcopenia or muscle functionality with precision. As a result, the functional benefits of the intervention could not be thoroughly evaluated.

Addressing these limitations in future studies through prospective designs, the inclusion of control groups, professional supervision, and the use of objective assessment tools will enhance the robustness of findings and provide deeper insights into the impact of combined dietary and physical activity interventions.

## 5. Conclusions

In conclusion, this study underscores the transformative role of physical activity in managing CKD and oncological conditions. Integrating structured physical activity with dietary management significantly improved physical performance, body composition, and metabolic health while maintaining renal function. Future research should focus on large-scale randomized controlled trials to refine tailored protocols and explore long-term impacts on survival, CKD progression, and cancer recurrence.

## Figures and Tables

**Table 1:** A descriptive statistical analysis of the cancer patient cohort (oncology) and the CKD control group (control) (**a**). Urological cancers and surgery in the oncology cohort (**b**). ^1^ Kruskal–Wallis rank sum test. ^2^ Pearson’s Chi-Squared test.

(**a**)					
**Variables**		**All**	**Oncology**	**Control**	***p*-Value**
Patients – n (%)		81	50 (61.7)	31 (38.3)	
Male sex – n (%)		62 (79.5)	41 (82.0)	21 (67.7)	0.14 ^2^
Age – years	Mean	70.9	71.9	67.9	0.44 ^1^
	Interquartile range	63–76	63.7–75.6	60.1–77.0	
Body mass index (kg/m^2^)	Median	25.99	26.0	25.3	0.97 ^2^
	Interquartile range	18.79–39.76	23.5–29.4	23.3–30.6	
Year of birth – n (%)	<1943	14 (17.3)	7 (14)	7 (22.6)	0.29 ^2^
	1943–1947	19 (23.4)	15 (30)	4 (12.9)	
	1948–1957	28 (34.6)	17 (34)	11 (35.5)	
	>1957	20 (24.7)	11 (22)	9 (29)	
BMI – n (%)	Healthy weight	36 (44.4)	22 (27.2)	14 (17.3)	0.52 ^2^
	Overweight	26 (32.1)	18 (22.2)	8 (9.9)	
	Obese	19 (23.5)	10 (12.4)	9 (11.1)	
CKD stage – n (%)	G2	7 (8.6)	3 (6.0)	4 (12.9)	0.06 ^2^
	G3a	29 (35.8)	22 (44.0)	7 (22.6)	
	G3b	20 (24.7)	13 (26.6)	7 (22.6)	
	G4	19 (23.4)	11 (22.0)	8 (25.8)	
	G5	6 (7.5)	1 (2.0)	5 (16.1)	
Diabetes – n (%)		9 (11.1)	7 (14.0)	2 (6.4)	0.49 ^2^
Hypertension – n (%)		62 (76.5)	37 (74.0)	25 (80.6)	0.68 ^2^
(**b**)					
**Tumor Location**	**Total**	**Surgery**			**Frequency**
Cancer type	55				
Kidney	26	Radical Nephrectomy			16
		Partial Nephrectomy			10
Prostate	6	Prostatectomy			6
Bladder	17	Cystectomy			14
		TURBK			3
Urothelium	6	Nephroureterectomy			4
		TURBK			2

**Table 2:** Univariate fixed effect analysis of RAPA2 score with RAPA 1 score. This table presents the univariate fixed effect analysis of RAPA2 as the dependent variable. Coefficients (estimate), standard errors, and 95% confidence intervals (CI) are provided.

Parameter	Estimate	Std. Error	Sig.	95% Confidence Interval	
				Lower Bound	Upper Bound
[RAPA2 = 0]	−1.539386	0.241465	0.000	−2.019745	−1.059027
[RAPA2 = 1]	−0.362526	0.263335	0.172	−0.886512	0.161460
[RAPA2 = 2]	−0.930268	0.341230	0.008	−1.607587	−0.252949
[RAPA2 = 3]	0	0	.	.	.

**Table 3:** Changes in body composition, hydration status, and cellular health parameters before and after the intervention. Values are presented as medians with interquartile ranges. Pearson’s Chi-Squared test. ECM/BCM: extracellular mass to body cell mass ratio; BCM/H^2^: body cell mass normalized to height squared; BMI: body mass index, calculated as weight (kg) divided by height squared (m^2^); ECW/ICW: extracellular water to intracellular water ratio; TBW: total body water; MAMC: mid-arm muscle circumference; FM/H^2^: fat mass normalized to height squared; FFM/H^2^: fat-free mass normalized to height squared.

Variable	Before [Range]	After [Range]	*p*-Value
BMI	25.99 [23.43–29.73]	25.46 [23.25–28.31]	0.0009 ***
Waist circumference	96.00 [85.00–105.00]	93.00 [85.00–100.00]	0.0005 ***
PhaseAngle_AP	5.70 [5.05–6.40]	5.90 [5.25–6.45]	0.0008 ***
ECM/BCM	0.91 [0.78–1.00]	0.87 [0.77–0.99]	0.0059 **
BCM/h^2^	11.06 [9.48–12.25]	11.46 [9.74–12.57]	0.0076 **
ECW/ICW	0.89 [0.78–1.01]	0.86 [0.74–0.97]	0.0005 ***
TBW	59.30 [54.15–61.80]	61.00 [56.20–63.35]	0.0013 **
MAMC	25.86 [24.40–27.73]	25.49 [23.97–27.66]	0.0714
FM/h^2^	5.25 [3.55–7.55]	3.95 [2.88–6.14]	0.0000 ***
FM/h^2^	21.05 [19.73–22.40]	21.35 [19.80–23.02]	0.0000 ***

** indicates statistical significance for *p* = 0.01 and *** for *p* = 0.001.

**Table 4:** Univariate fixed effect analysis of RAPA1 with physiological parameters. This table presents the univariate fixed effect analysis of RAPA1 as the dependent variable. Parameters include phase angle (PA), body mass index (BMI), waist circumference, total body water percentage (TBW%), extracellular mass to body cell mass ratio (ECM/BCM), body cell mass adjusted for height (BCM/h^2^), extracellular to intracellular water ratio (ECW/ICW), fat mass adjusted for height (FM/h^2^), fat-free mass adjusted for height (FFM/h^2^), and mid-arm muscle circumference (MAMC). Coefficients (estimate), standard errors, and 95% confidence intervals (CI) are provided. Statistically significant predictors of RAPA1 include PA, TBW%, ECM/BCM, BCM/h^2^, ECW/ICW, FM/h^2^, FFM/h^2^, and MAMC (*p* < 0.05), with non-significant results noted for BMI and waist circumference.

					95% Confidence Interval	
Parameter	Estimate	Std. Error	t-Value	*p*-Value	Lower Bound	Upper Bound
PA	0.851	0.087	9.716	<0.001	0.676	1.026
BMI	−0.020	0.028	−0.705	0.482	−0.077	0.036
Waist Circumference	−0.007	0.009	−0.731	0.466	−0.026	0.012
TBW%	0.072	0.017	4.122	<0.001	0.037	0.107
ECM/BCM	−4.846	0.590	−8.207	<0.001	−6.020	−3.671
BCM/h^2^	0.321	0.054	5.877	<0.001	0.212	0.429
ECW/ICW	−3.943	0.493	−7.995	<0.001	−4.924	−2.961
FM/h^2^	−0.140	0.035	−3.971	<0.001	−0.211	−0.070
FFM/h^2^	0.184	0.045	4.042	<0.001	0.094	0.275
MAMC	0.095	0.042	2.24	0.028	0.010	0.179

**Table 5:** Summary of changes in clinical parameters before and after the intervention. Values are presented as medians with interquartile ranges (IQR). The *p*-values were calculated using the Wilcoxon signed-rank test for paired data.

Variable–Median [IQR]	Before	After	*p*-Value
Serum creatinine	1.615 [1.41–2.1375]	1.58 [1.38–2.31]	0.46375
eGFR (CKD-EPI 2021)	44.0 [33.0–52.25]	45.0 [37.0–47.0]	0.74356
mGFR (Iohexol)	35.155 [24.87–42.17]	35.5 [24.82–44.82]	0.74356
Serum urea	61.5 [42.75–75.5]	48.5 [40.5–66.0]	0.00516

**Table 6:** Multivariate fixed effect analysis of RAPA1 with nephrological parameters. This table presents the multivariate effect analysis of RAPA1 as the dependent variable. Independent variables include eGFR, Iohexol, Serum creatinine, and Cystatin C.

					95% Confidence Interval	
Parameter	Estimate	Std. Error	t-Value	*p*-Value	Lower Bound	Upper Bound
Intercept	−0.609	1.443	−0.422	0.675	−3.521	2.302
eGFR	−0.005	0.011	−0.441	0.661	−0.027	0.017
mGFR (Iohexol)	0.06	0.019	3.028	0.004	0.020	0.100
Serum Creatinine	0.079	0.248	0.321	0.751	−0.429	0.588

## Data Availability

All the data are stored in institutional repositories, further inquiries can be directed to the corresponding author.

## Supplementary Materials

The following supporting information can be downloaded at: https://www.mdpi.com/article/10.3390/nu17020335/s1. Table S1. Univariate fixed effect analysis of RAPA1 with physiological parameters. This table presents the univariate fixed effect analysis of RAPA1 as the dependent variable. Parameters include phase angle (PA), body mass index (BMI), waist circumference, total body water percentage (TBW%), extracellular mass to body cell mass ratio (ECM/BCM), body cell mass adjusted for height (BCM/h^2^), extracellular to intracellular water ratio (ECW/ICW), fat mass adjusted for height (FM/h^2^), fat-free mass adjusted for height (FFM/h^2^), and mid-arm muscle circumference (MAMC). Coefficients (estimate), standard errors, and 95% confidence intervals (CI) are provided. Statistically significant predictors of RAPA1 include PA, TBW%, ECM/BCM, BCM/h^2^, ECW/ICW, FM/h^2^, FFM/h^2^, and MAMC (*p* < 0.05), with non-significant results noted for BMI and waist circumference.
