# Supplementary material for: The Role of Maintaining Nutritional Adequacy Status and Physical Activity in Onco-Nephrology: Not a Myth Anymore, but a Reality
Source: Nutrients. 2025 Jan 17;17(2):335. doi: 10.3390/nu17020335 (PMC11768965; doi:10.3390/nu17020335)
Supplement: Supplementary file 1 [file nutrients-17-00335-s001.zip › nutrients-3365950-supplementary.pdf]

Table S1. Univariate Fixed Effect Analysis of RAPA1 with Physiological Parameters. This table presents the univariate fixed effect analysis of RAPA1 as the dependent variable. Parameters include phase angle (PA), body mass index (BMI), waist circumference, total body water percentage (TBW%), extracellular mass to body cell mass ratio (ECM/BCM), body cell mass adjusted for height (BCM/h^2), extracellular to intracellular water ratio (ECW/ICW), fat mass adjusted for height (FM/h^2), fat-free mass adjusted for height (FFM/h^2), and mid-arm muscle circumference (MAMC). Coefficients (Estimate), standard errors, and 95% confidence intervals (CI) are provided. Statistically significant predictors of RAPA1 include PA, TBW%, ECM/BCM, BCM/h^2, ECW/ICW, FM/h^2, FFM/h^2, and MAMC ($p < 0.05$), with non-significant results noted for BMI and waist circumference.

A)

				95% Confidence Interval	
**Parameter**	**Estimate**	**Std. Error**	**P-Value**	**Lower Bound**	**Upper Bound**
Intercept	-2.284334	.517345	.000***	-3.315993	-1.252675
Phase Angle_PA	.851645	.087653	.000***	.676874	1.026416

B)

				95% Confidence Interval	
**Parameter**	**Estimate**	**Std. Error**	**P-Value**	**Lower Bound**	**Upper Bound**
Intercept	3.284922	1.294385	.011**	.744089	5.825754
BMI	-.020374	.028893	.482	-.077695	.036947

C)

				95% Confidence Interval	
**Parameter**	**Estimate**	**Std. Error**	**P-Value**	**Lower Bound**	**Upper Bound**
Intercept	3.419984	.933010	1.000	-1121.569106	1128.409074
Waist Circumference	-.007164	.009800	.466	-.026610	.012281

D)

				95% Confidence Interval	
**Parameter**	**Estimate**	**Std. Error**	**P-Value**	**Lower Bound**	**Upper Bound**
Intercept	-1.464109	1.158821	.208	-3.752058	.823839
TBW%	.072684	.017634	.000***	.037743	.107624

E)

				95% Confidence Interval	
**Parameter**	**Estimate**	**Std. Error**	**P-Value**	**Lower Bound**	**Upper Bound**
Intercept	7.119275	.853597	.000***	5.442303	8.796248
ECM/BCM	-4.846064	.590482	.000***	-6.020171	-3.671957

F)

				95% Confidence Interval	
**Parameter**	**Estimate**	**Std. Error**	**P-Value**	**Lower Bound**	**Upper Bound**
Intercept	-.861940	.627515	1.000	-216.972023	215.248143
BCM/h2	.321152	.054641	.000***	.212599	.429704

G)

				95% Confidence Interval	
**Parameter**	**Estimate**	**Std. Error**	**P-Value**	**Lower Bound**	**Upper Bound**
Intercept	6.301143	.460303	.000***	5.385345	7.216941
ECW/ICW	-3.943194	.493211	.000***	-4.924440	-2.961948

H)

				95% Confidence Interval	
**Parameter**	**Estimate**	**Std. Error**	**P-Value**	**Lower Bound**	**Upper Bound**
Intercept	3.539229	.223488	.000***	3.095939	3.982518
FM/h2	-.140967	.035501	.000***	-.211374	-.070560

I)

				95% Confidence Interval	
**Parameter**	**Estimate**	**Std. Error**	**P-Value**	**Lower Bound**	**Upper Bound**
Intercept	-1.164613	1.308337	1.000	-12.454244	10.125018
FFM/h2	.184884	.045745	.000***	.094099	.275670

L)

				95% Confidence Interval	
**Parameter**	**Estimate**	**Std. Error**	**P-Value**	**Lower Bound**	**Upper Bound**
Intercept	.400810	1.101822	.717	-1.789620	2.591240
MAMC	.095358	.042573	.028**	.010718	.179998
